# EXPLORING THE ASSOCIATION BETWEEN FAMILY DYNAMICS AND PREPAREDNESS FOR FAMILY CAREGIVING AT THE END OF LIFE

**DOI:** 10.1093/geroni/igac059.400

**Published:** 2022-12-20

**Authors:** Aimee Fox, Julia Sharp, Christine Fruhauf

**Affiliations:** Kansas State University, Manhattan, Kansas, United States; Colorado State University, Fort Collins, Colorado, United States; Colorado State University, Fort Collins, Colorado, United States

## Abstract

The transition to providing end-of-life (EOL) care to a family member can be challenging for family caregivers as they face difficult decisions regarding medical course of treatment, increasing caregiving responsibilities, and anticipatory grief. Low preparedness for EOL caregiving is associated with higher levels of caregiver strain, increased levels of depression and anxiety, and complicated and prolonged grief after the death of the family member. Despite the breadth of caregiving research, little is known about how family relationships and interactions relate to caregiver preparedness for EOL caregiving. Thus, the purpose of this study was to explore the association between family caregivers’ family dynamics and their perceived preparedness for the transition to EOL caregiving. A sample of 173 family caregivers were recruited to complete an online, self-report survey. A structural equation model was used to analyze the association between family dynamics and caregiving preparedness. Most caregivers reported balanced family cohesion (75.1%) and family flexibility (75.7%), but low family communication and low family satisfaction. In addition, 20.2% of caregivers reported being not at all prepared for the transition to EOL caregiving. Although there was a lack of meaningful association between family dynamics and preparedness for EOL caregiving, it may be that family relationships and interactions grow more complex as families age and individuals take on new and different roles (such as family caregiving) within the family system. This study demonstrates the need for future research to develop new measures to explore how aging family dynamics relate to family caregiver experiences and outcomes.

